# Pediatric unicystic cystic partially differentiated nephroblastoma: The first case report

**DOI:** 10.3389/fped.2022.1045936

**Published:** 2022-11-29

**Authors:** Liu Chao, Zhang Lei, Li Xiang, Zhou Qi

**Affiliations:** Department of Pediatric Surgery, Qilu Hospital, Shandong University, Jinan, China

**Keywords:** case report, pediatric, unicystic, cystic partially differentiated nephroblastoma, first

## Abstract

Cases of patients with cystic partially differentiated nephroblastoma (CPDN) have been reported to date, which presented as polycystic renal tumor in all of them. It is a special pathological type of nephroblastoma. Here, we report the first case of a unicystic CPDN in a child. The patient was diagnosed with a simple renal cyst and underwent laparoscopic decortication. The naive nephron was found in the pathological section, and the diagnosis of CPDN was confirmed. The patient then underwent a radical nephrectomy and six cycles of postoperative chemotherapy. There was no recurrence or metastasis after 2 years of follow-up. Pediatric CPDN presenting as a unicystic renal tumor poses a new challenge to the diagnosis, differential diagnosis, and treatment of unicystic renal tumor.

## Introduction

Cystic partially differentiated nephroblastoma (CPDN) usually occurs in children under 2 years old, with a male-to-female ratio of about 2:1 (1, 2). It is a rare type of nephroblastoma, which is recognized as a tumor with low malignant potential (3). More than 120 cases of patients with CPDN have been reported to date, and all of them had the gross/macroscopic appearance of a polycystic renal tumor, previously considered to be the only pathological presentation. Clinical features and image studies have failed to discriminate CPDN from cystic nephroma (CN) and cystic Wilms tumor (CWT) (4). Here, we present the first case of pediatric CPDN presenting as a unicystic renal tumor and propose new insights into the differential diagnosis and treatment of unicystic renal tumor.

## Case presentation

**Figure 1:**
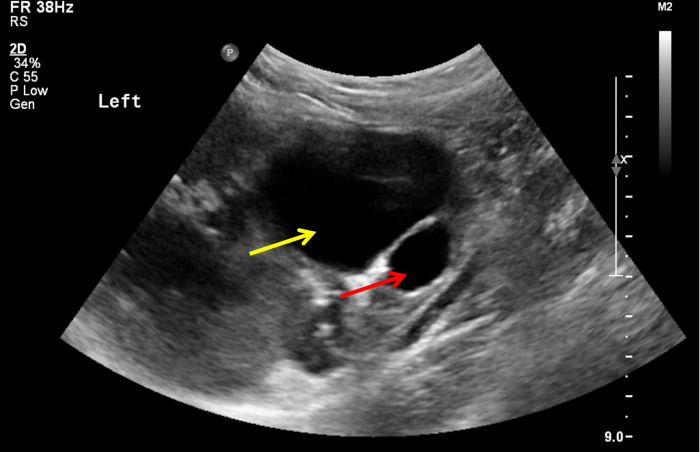
Image of ultrasound: unicystic tumor (yellow arrow) in the left kidney with a clear boundary and uniform internal sound transmission, accompanied by a dilatation of the renal pelvis (red arrow).

**Figure 2:**
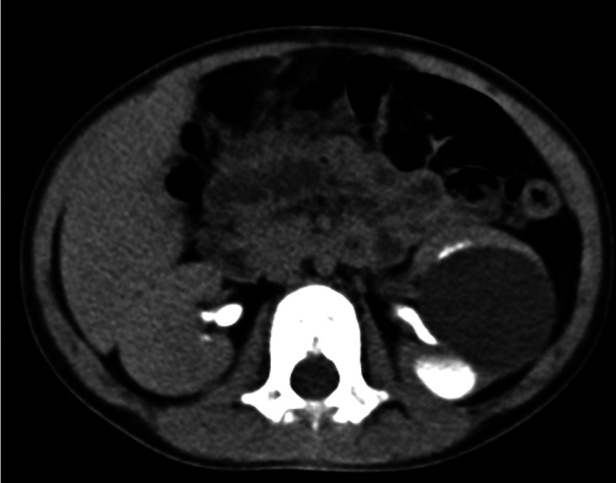
Image of an abdominal enhanced CT: unicystic tumor in the left kidney with a clear boundary but without contrast filling. There was contrast filling in the renal pelvis.

A 3-year-old girl was diagnosed with a cystic tumor on the left kidney by abdominal ultrasound in another hospital 2 weeks prior after she experienced occasional abdominal pain, but she had no abdominal pain when she presented to our clinic. The reexamined ultrasound showed a cystic tumor inside the left kidney, with a size of 4.6 × 3.9 cm, with a clear boundary, thin wall, and uniform internal sound transmission (Figure 1, yellow arrow). An abdominal enhanced CT showed a cystic low-density lesion inside the left kidney with a clear boundary, about 4.0 × 3.8 × 3.5 cm in size, and without contrast filling. The adjacent renal pelvis was dilated with a diameter of about 1.9 cm, and there was contrast filling in the renal pelvis (Figure 2).

A measurement of the patient’s kidney showed a creatinine level of 33 µmol/L and a urea nitrogen level of 4.6 mmol/L. Tumor marker tests showed carcinoembryonic antigen (CEA) <0.30 ng/ml, alpha fetoprotein (AFP) 2.15 IU/ml, carbohydrate antigen 125 (CA-125) 17.0 U/ml, carbohydrate antigen 19-9 (CA19-9) 9.42 U/ml, β-human chorionic gonadotropin (β-HCG) < 0.20 mIU/ml, neuron-specific enolase (NSE) 11.6 ng/ml, and cytokeratin 19 fragment (CK-19) 1.8 ng/ml. However, the girl’s routine blood test and urinalysis were normal.

**Figure 3:**
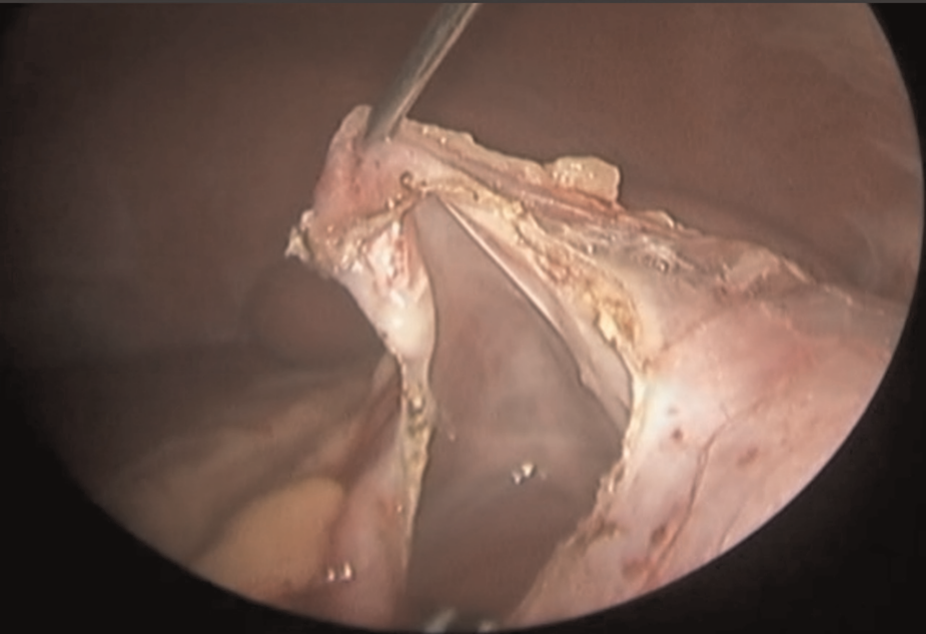
Image of a laparoscopic decortication of the renal cyst. After incision of the cyst wall, the inner wall of the cyst became smooth, and the cyst became unicystic.

**Figure 4:**
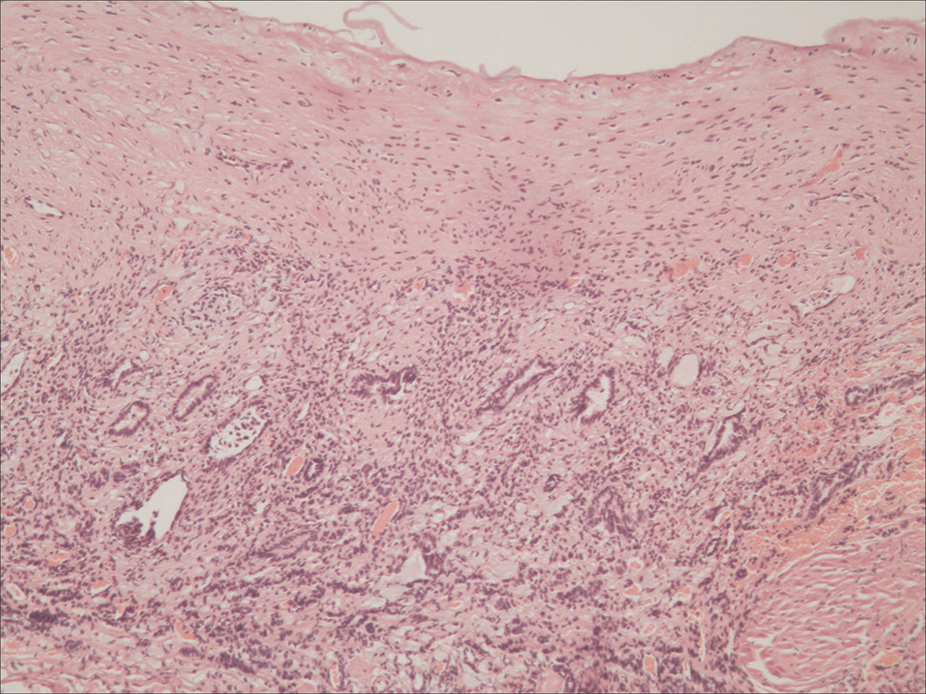
Image of histopathological examination (HE, ×100). The naive nephron was found in the pathological section.

The patient was diagnosed with a simple left renal cyst, and she underwent a laparoscopic decortication of the renal cyst. During the operation, a unicystic tumor in the middle and upper pole of the left kidney was revealed, the inner wall of the cyst was smooth, the cystic fluid was clear, and there was no obvious connection between the cyst and the renal pelvis (Figure 3). The cyst wall with an area of 3 × 3 cm was resected. Immature nephrons of different sizes/stages of differentiation were found in the histological sections (Figure 4). The diagnosis of CPDN was confirmed.

In the second operation, a radical nephrectomy was performed on the left kidney, and the operated area was rinsed with distilled water. No abnormal lymph nodes were found. The second histopathological examination also showed CPDN, with the immunohistochemistry showing vimentin (+), Wilms tumor protein (+), epithelial membrane antigen (+), acidic calcium-binding protein S-100 (−), neuron-specific enolase (−), and a protein Ki-67 index of 17%.

Six cycles of chemotherapy were performed after the operation, and the chemotherapy protocol was vincristine combined with dactinomycin. There was no recurrence of the tumor and no occurrence of metastasis after 2 years of follow-up.

## Discussion

Brown standardized the naming of CPDN for the first time in 1975 (5). He suggested that CNPD was a low-grade malignant or malignant renal cystic tumor and that WT could undergo spontaneous maturation to a fully differentiated, benign, and cystic tumor. Previous studies have suggested that WT, CPDN and CN represent different degrees of differentiation in the same differentiation spectrum, with WT being the least differentiated and CN being the most differentiated ends of the spectrum and CPDN being the transition between the two (6).

A complete search of the PubMed, EMBASE, and Wanfang databases was performed in the period between January 1975 and May 2022 to identify all reports describing pediatric CPDN. We also included articles that described CPDN, as this is the most important differential diagnosis in children with CWT and CN. A cross-reference check was used to identify potential additional reports. For multiple case reports from the same author or institution, we checked each case carefully to avoid the same case being repeatedly included in the study. Cases with a high probability of repetition were also excluded from the study. The inclusion criteria of the articles included the following conditions: First, the article had to report well-described pediatric patients with CPDN. Second, the article had to be an original one. Third, the article had to be available as a full text or a well-described abstract. Fourth, the article had to be written in English, Spanish, Chinese, or Dutch. All manuscripts were screened by two independent reviewers (Liu Chao and Li Xiang). A total of 121 cases of pediatric CPDN were retrieved from 48 articles. In all cases, the tumors were polycystic, as described by surgical specimen pictures or literature. A polycystic renal tumor was considered the only manifestation of CPDN (7). However, the case that we report in this study is a patient with an unicystic renal tumor, which is the first of its kind.

CPDN has no specific clinical characteristics, and most patients show only a painless abdominal mass found unintentionally or a renal tumor found by abdominal ultrasound or CT (8). In the statistical literature, abdominal mass accounted for 87.1% (54/62) of cases. Our present case is a patient with a unicystic renal tumor inadvertently found by ultrasonography.

At present, establishing the correct diagnosis of CPDN requires a careful histopathological examination of the resected tumor because imaging studies have failed to discriminate among CPDN, CN, and CWT (9). In 1998, Eble and Bonsib proposed a complete diagnostic method for CPDN (1). The patients in their study were mainly children under 2 years old. The tumor is an expansive mass surrounded by a fibrous pseudocapsule, and the interior of the tumor is fully composed of cysts and septa, without expansive solid nodules. The cyst is lined by a flattened, hobnail, or cuboidal epithelium. The septa may contain epithelial structures resembling mature renal tubules and may also contain blastema, with or without embryonic stromal or epithelial elements.

Our patient was initially misdiagnosed as having a simple renal cyst because of the unicystic manifestation of the tumor, but the final histopathological examination confirmed the diagnosis of CPDN. The unicystic manifestation of CPDN not only enriches our understanding of CPDN but also poses a new challenge to the diagnosis and differential diagnosis of unicystic renal tumor. We consider that some of the diagnostic criteria proposed by Eble and Bonsib should be further improved. The tumor is composed of single or multiple cysts, without solid expandable nodules, with or without septum. The septum or cyst wall may contain epithelial structures that are immature or resemble mature renal tubules.

For polycystic renal tumors such as CN, CPDN, and CWT, tumorectomy or nephrectomy is the most common surgical method, and biopsies to confirm the diagnosis are not encouraged (10–12). Our study involved an examination of the kidneys of 125 patients, of which 119had polycystic CPDN, and out of the 125, 86 patients underwent nephrectomy, 15 underwent partial nephrectomy, and information on the remaining 24 was not available. However, these surgical methods may not be completely applicable to unicystic renal tumor because the incidence of simple renal cysts is much higher than that of unicystic CPDN, and simple renal cysts are usually operated upon by a decortication of the renal cyst (13).

We considered that partial tumorectomy or a decortication of the renal cyst can still be used preferentially for unicystic renal tumors, without excluding the possibility of CPDN, but the role of the intraoperative frozen section during surgery should be emphasized (14). Although tumorectomy or decortication of the renal cyst may lead to a rupture of CPDN, resulting in a change in the tumor stage from stage I to stage III, we can further treat it by washing the operated area with distilled water and postoperative chemotherapy. The patient we reported received intraoperative distilled water washing and six cycles of chemotherapy after the operation, and there was no recurrence of the tumor or occurrence of metastasis after 2 years of follow-up, which may be related to the low malignancy and low invasiveness of CPDN.

According to the conclusions provided by the International Society of Pediatric Oncology (SIOP), preoperative chemotherapy cannot effectively reduce the size of a CPDN tumor (9). The 10 CPDN patients examined in the study were treated with preoperative chemotherapy, which in all cases did not reduce the size of the tumor. As recommended by the National Wilms Tumor Study Group (NWTSG), for stage I CPDN, only surgical resection is required. However, for stage II and stage III CPDN, the tumor cannot be resected completely, and postoperative chemotherapy is considered effective (15). In the statistical literature, it has been reported that 47 patients received postoperative chemotherapy. Although a few patients had different manifestations of chemotherapy complications, there was no death directly related to chemotherapy. Therefore, postoperative chemotherapy is recommended for intraoperative tumor rupture caused by a misdiagnosis of CPDN as a simple renal cyst or other benign tumors.

The postoperative recurrence rate of CPDN was low (4). The 92 patients examined in the study were reported for follow-up, including 3 patients with local recurrence and 1 with CWT recurrence after undergoing partial nephrectomy for bilateral CPDN. Chemotherapy was given after recurrence for these 4 patients, and the survival rate was good during the follow-up period. There were no deaths directly related to CPDN or recurrence (4).

In summary, CPDN is rarely reported in the clinic and can be easily confused with CN and CWT. In this study, a unicystic pediatric CPDN is reported for the first time, which poses a new challenge to the diagnosis and treatment of renal cystic tumor. There are no specific clinical characteristics of pediatric CPDN. Surgery is the first choice of treatment, and histopathological examination is the only means of diagnosis. For patients with stage II CPDN or above, postoperative chemotherapy is recommended. The recurrence rate of CPDN is low, and the overall prognosis is good.

## Data Availability

The datasets presented in this study can be found in online repositories. The names of the repository/repositories and accession number(s) can be found in the article/Supplementary Material.
